# Adjuvant bleomycin, vincristine and cisplatin (BOP) for high-risk stage I non-seminomatous germ cell tumours: a prospective trial (MRC TE17)

**DOI:** 10.1038/sj.bjc.6602624

**Published:** 2005-05-31

**Authors:** D P Dearnaley, S D Fossa, S B Kaye, M H Cullen, S J Harland, M P J Sokal, J D Graham, J T Roberts, G M Mead, M V Williams, P A Cook, S P Stenning

**Affiliations:** 1 Academic Radiotherapy, Institute of Cancer Research and Royal Marsden Hospital, Sutton, Surrey SM2 5PT, UK; 2 Norwegian Radium Hospital, Oslo 0310, Norway; 3 Beatson Oncology Centre, Glasgow G11 6NT, Scotland; 4 University Hospital Birmingham NHS Foundation Trust, Birmingham B15 2TH, UK; 5 The Meyerstein Institute of Oncology, The Middlesex Hospital, Mortimer Street, London W1N 8AA, UK; 6 Nottingham City Hospital, Hucknall Road, Nottingham NG5 1PB, UK; 7 Bristol Haematology and Oncology and Centre, Bristol BS2 8ED, UK; 8 Northern Centre for Cancer Treatment, Newcastle-upon-Tyne NE4 6BE, UK; 9 Southampton General Hospital, Southampton S016 6YD, UK; 10 Oncology Department, Addenbrooke's Hospital, Cambridge CB2 2QQ, UK; 11 MRC Clinical Trials Unit, 222 Euston Road, London NW1 2DA, UK

**Keywords:** adjuvant chemotherapy, stage I non-seminoma

## Abstract

Adjuvant BEP (bleomycin, etoposide, cisplatin) is effective treatment for high-risk clinical stage I (HRCS1) non-seminomatous germ cell tumours (NSGCT), but the known toxicities of etoposide, and the expansion of the HR group to any patient with vascular invasion (50% of patients), led the Medical Research Council to pilot the BOP regimen. Patients received two courses of BOP 14 days apart: cisplatin 50 mg m^−2^ days 1 and 2, vincristine 1.4 mg m^−2^ (max. 2 mg) days 2 and 8, bleomycin 30 000 IU days 2 and 8. Primary outcome was relapse rate; quality of life, fertility, hearing and lung function were assessed pre- and post-treatment. In all, 100 patients were required. A total of 115 eligible patients were registered, all received two courses of chemotherapy. Median follow-up is 70 months; two relapses have occurred and the 5-year relapse-free rate is 98.3% (95% confidence interval (CI) 95.5%, 99.9%). As assessed by clinicians during treatment, complete (reversible) alopecia was present in 20% of patients; World Health Organization (WHO) grade 1/2 neurotoxicity was present in 41%/5% of patients during treatment and 22%/1% at 6 months. However, 12% of patients reported ‘quite a bit’ or ‘very much’ pain/numbness/tingling in hands/feet 2 years after chemotherapy. Mature follow-up confirms high efficacy for two courses of cisplatin-based adjuvant chemotherapy in HRCS1 NSGCT. Substituting vincristine for etoposide decreases alopecia, but gives a low incidence of significant neuropathy. There are no clearcut advantages to 2 × BOP over 2 × BEP, except for patients who wish to maximise the chance of avoiding significant alopecia.

Approximately 40% of men with non-seminomatous germ cell tumours (NSGCT) of the testis present with stage I disease. Surveillance has become a widely used initial management option for these patients, following the successful treatment of disseminated germ cell tumours with cisplatin-based chemotherapy. Two Medical Research Council (MRC) studies of surveillance (Freedman *et al*, 1987; Read *et al*, 1992) have shown that, overall, approximately 25–30% of men with clinical stage I NSGCT develop metastatic disease but that histopathological risk factors (invasion of the testicular veins and/or lymphatics, absence of yolk sac elements and presence of undifferentiated teratoma) could be used to identify a high-risk group. Just over 20% of patients had three or all four of these features, and their 2-year relapse-free rate was 53%, compared with 83% in those with two or fewer risk factors.

This risk was considered sufficiently high to warrant immediate treatment, and the MRC TE05 trial (Cullen *et al*, 1996a) assessed the value of two cycles of standard cytotoxic chemotherapy (BEP: bleomycin, etoposide and cisplatin) in preventing recurrence. Data published with a median follow-up of 4 years showed only one relapse in the 103 patients with confirmed germ cell tumour on review. These results suggested that it may be appropriate to offer a broader range of patients adjuvant treatment.

Further analysis of the second MRC surveillance study (Read *et al*, 1992) together with examination of the intra- and interpathologist variation in identifying risk factors established that venous and lymphatic invasion were not always easily distinguishable. However, the presence of either, irrespective of other risk factors, conferred a risk of relapse of 35–40%. Approximately 50% of stage I patients have vascular invasion and, in view of the fact that around 60% of these ‘high-risk’ patients are not destined to relapse, the MRC Testis Tumour Working Party considered whether there were alternative regimens that might reduce the side effects associated with BEP. Although these are relatively modest after two courses, side effects include total alopecia, myelosuppression, reduced fertility and renal and auditory dysfunction.

Etoposide is the drug principally responsible for alopecia and myelosuppression, and possibly leukaemogenesis in a dose-related manner. Therefore a logical step was to consider regimens without etoposide. The present study evaluated the BOP regimen with respect to short- and long-term toxicity and relapse rate.

Despite limited data on the efficacy of vincristine as a single agent, schedules replacing etoposide by vincristine have been shown to be effective (Merrin, 1980; Newlands *et al*, 1983; Wettlaufer *et al*, 1984; Dearnaley, 1991; Lewis *et al*, 1991; Christian *et al*, 2003). As BOP schedules are less myelosuppressive than BEP, the treatment frequency can be increased and courses given at 1–2 week intervals, which may be advantageous in the treatment of rapidly proliferating tumours (Dearnaley, 1991). The treatment time of each cycle of adjuvant BOP was reduced to 48 h, compared to 3 days or more for BEP treatment, and the overall duration of treatment reduced from 5 weeks with BEP to 3 weeks. Vincristine at standard dose causes little if any alopecia and it was therefore expected that the BOP regimen would not be associated with significant alopecia.

The MRC therefore initiated a multicentre phase II pilot study of two courses of adjuvant BOP, including assessment of toxicity, relapse rate and quality of life (QoL). If this or a subsequent regimen was shown to have a good toxicity profile, and no clear evidence of inferior relapse rates was identified, a randomised trial against the standard BEP could be considered.

## PATIENTS AND METHODS

### Eligibility

Patients had histologically confirmed NSGCT (included mixed tumours) with high-risk features (tumour invasion of the testicular lymphatics and or the testicular veins), and radiologically and clinically confirmed stage I disease with normal tumour markers (AFP, HCG) following orchidectomy and normal renal function (creatinine clearance >75 ml min^−1^). Patients were to be registered within 6–8 weeks of orchidectomy, or within 4 weeks of postorchidectomy marker normalisation. Patients with elevated markers, which did not fall at the rate expected from the marker half-life were not eligible. The trial protocol was approved by each participating centre's research ethics committee, and all patients gave informed consent to be in the trial.

Patients were registered on study, prior to commencing chemotherapy, by a telephone call to the MRC Cancer Trials Office, Cambridge UK (now the MRC Clinical Trials Unit, Cancer Division).

### Treatment

Within strict dose constraints, clinicians were able to employ the schedule, fluid and antiemetic regimen with which they were most familiar. Between days 1 and 2, 100 mg m^−2^ of cisplatin, 1.4 mg m^−2^ (max. 2 mg) of vincristine and 30 000 IU of bleomycin were given. The same doses of vincristine and bleomycin were repeated on day 8. The cycle was repeated on day 15 at full doses if the white cell count (WCC) was ⩾2.0 × 10^9^ l^−1^, the platelet count was ⩾100 × 10^9^ l^−1^ and either serum creatinine was <120 mmol l^−1^ or renal clearance >60 ml min^−1^. If on day 15 the WBC <2 × 10^9^ l^−1^ or platelets <100 × 10^9^ l^−1^, blood counts were repeated daily until they exceeded these levels, at which time the cycle started at full dose. If day 15 renal clearance was between 45 and 60 ml min^−1^, 50% cisplatin dose was given; treatment at lower renal clearance rates was on the advice of the clinical coordinator.

### Postchemotherapy follow-up

Patients attended for follow-up at monthly intervals for the first year, 2-monthly in the second year, three-monthly in the third year and at 6-monthly intervals to 5 years. Subsequent follow-up was according to usual centre practice. At each visit, a clinical examination was performed, and blood taken for AFP and HCG. Chest X-rays were performed at least every 2 months in the first year, and chest and abdomen/pelvis CTs were performed at 6, 12 and 24 months from the start of chemotherapy. In the event of recurrence, treatment was given according to the centres preferred regimen for poor risk/recurrent metastatic teratoma.

### Outcome measures

#### Relapse rate

Time from start of chemotherapy to relapse, or date last seen.

#### Acute toxicity

Two weeks after cycle 2, clinicians reported the maximum World Health Organization (WHO) grade of alopecia, neurotoxicity, renal, mucosal and pulmonary toxicity and nausea/vomiting since the start of chemotherapy. Alopecia was further assessed at 2, 3 and 4 months after the start of chemotherapy, while neurotoxicity was assessed at 3, 6, 12 and 24 months.

#### Long-term toxicity

Prior to commencing chemotherapy sperm analysis, hormone (LH, FSH, testosterone) analysis, audiometry and pulmonary function tests were performed. These were repeated at 12 months, and again at 2 years from the start of chemotherapy.

#### Quality of life

Health-related QoL was assessed through the use of the EORTC core questionnaire (QLQ-C30) and the testicular cancer module then under development (Fossa *et al*, 1996). Patients were asked to complete the baseline questionnaires in the clinic; the Trials Office then sent subsequent forms directly to the patients' homes, with pre-paid, preaddressed return envelopes, approximately one week before the due completion dates of 4 weeks, 6 months, 12 months and 24 months after starting chemotherapy.

### Statistical considerations

In all, 100 patients were to be entered into the trial, enabling the relapse rate to be estimated with an s.e. error <5%. As a relapse rate of more than 5% would be considered unacceptable, the data were monitored using an early termination scheme in which the probability that the final relapse rate would exceed 5% was calculated, conditional on the current data, at the time of each relapse, as in our previous study (Cullen *et al*, 1996a).

Relapse-free rates were estimated using the Kaplan–Meier method. Toxicity differences pre- and post-treatment were compared using either the Wilcoxon test for matched pairs, or the paired *t*-test if normality could be assumed.

The QLQ-C30 was scored according to the EORTC Scoring Manual (EORTC, 1995). This identifies five functional scales (physical, role, cognitive, social and emotional), one global health scale, three multi-item symptom scales (fatigue, nausea and vomiting, and pain), five individual symptom items (dyspnoea, insomnia, appetite loss, constipation, and diarrhoea) and a question on financial difficulties. Each scale is standardised to range in score from 0 to 100.

The testicular tumour questionnaire contains 20 questions which were analysed individually. Only forms completed within acceptable windows were used. Pretreatment forms had to be completed on or before the day chemotherapy started; end-of-treatment forms 4–8 weeks after the start of chemotherapy; 6-month forms 5–7 months postchemotherapy, 12-month forms 10–14 months postchemotherapy and 24-month forms 22–26 months postchemotherapy.

The analysis focuses on descriptive statistics, but where formal comparisons have been made of changes at specific time points relative to baseline scores, an extreme level of statistical significance (*P*<0.001) has been used to allow for the multiple comparisons being performed. The formal tests of within-patient changes from baseline used the Wilcoxon matched-pairs analysis.

## RESULTS

Between August 1994 and July 1996, 116 patients were registered from 16 centres in the UK and one in Norway. One patient who was found to have raised markers on the day chemotherapy was due to start was withdrawn and received instead three cycles of BEP (he remains relapse-free 6 years after registration). The 115 remaining patients form the basis of this report.

### Relapses

The median follow-up of relapse-free patients is 70 months. Two confirmed relapses have occurred. One patient relapsed with a 2 cm abdominal mass and negative markers 6 months after the start of chemotherapy. He received two courses of VIP prior to stem cell harvest and high-dose chemotherapy. He died from toxicity 3 months after diagnosis of his relapse. The second patient relapsed 3 months from the start of BOP with normal markers, a 3 cm abdominal mass and a small (<1 cm) lung mass. He received POMB/ACE, but progressed before completing treatment. He went on to further salvage including VIP (etoposide, ifosfamide and cisplatin) and docetaxel, and is now alive and disease-free 5 years after his first relapse was diagnosed. The 5-year relapse-free rate is 98.3% with 95% confidence interval (CI) (95.5%, 99.9%).

A third patient was suspected of having a relapse, with cerebral metastases but negative markers and no other sign of disease, 26 months after BOP. He received chemotherapy (VIP) with no impact on the brain CT appearances. Subsequent review and biopsy indicates that the lesions seen were inflammatory. He remains alive and free from any evidence of progression a further 2 years after the suspected relapse was seen.

### Chemotherapy compliance

All patients received two cycles of chemotherapy. Eight patients had intervals of more than 14 days between courses: two due to administrative errors, two due to haematological toxicity, one because of a sore throat and earache, and one due to tinnitus and declining pulmonary function.

No dose reductions of cisplatin were necessary. Three patients had dose reductions of vincristine in cycle 2 – one received none because of abdominal pain, one received only one day because of bleomycin-related toxicity (hyperpyrexia) and one had a 25% reduction due to neuropathy.

Four patients had dose reductions of bleomycin – one received only the first bleomycin dose of cycle 1 and none in cycle 2 because of a skin rash, one received no bleomycin in cycle 2 because of declining pulmonary function and two received only one dose in cycle 2 – one because of abdominal pain (as above) and one because of an unspecified bleomycin reaction.

### Clinician-assessed acute toxicity

The clinicians' assessment of toxicity at the end of chemotherapy is given in Table 1. No WHO grade 3 or 4 haematological toxicity was reported. Of 105 patients assessed at 6 months after the start of chemotherapy neurotoxicity was absent in 81 (77%), grade 1 in 23 (22%) and grade 2 in 1 (1%). Of 75 patients assessed at 12 months, 67 (89%) had none and the remaining eight (11%) had only grade 1 neurotoxicity. At 4 months after starting chemotherapy, alopecia was reported as absent or minimal in 79%, but still complete in 7%.

### Long-term toxicity

Table 2 details the long-term toxicity data. Only patients with pretreatment and follow-up assessments carried out at 1 year (±3 months) after chemotherapy are included.

In all, 37 patients had complete audiometry data sets. The results indicate a median 5 dB hearing loss at 8 kHz, which was statistically significant (*P*=0.008).

Between 22 and 29 patients (depending on the test performed) had complete data sets on pulmonary function tests pre- and post-treatment. There was no clear evidence of clinically relevant changes, although the mean 5% reduction in KCO reached conventional levels of statistical significance (*P*=0.03).

In 61 patients with complete data on renal clearance, the mean (s.d.) GFR fell from 124 (36) ml min^−1^ pretreatment to 113 (25) ml min^−1^ on follow-up. The estimated difference of 11 has a 95% CI (2–19%), *P*=0.014.

In the 39 patients with semen analysis carried out pretreatment and at 1 year, the median sperm density was 15 × 10^6^ ml^−1^ (interquartile range (IQ) range 9–30) before chemotherapy with 11 patients (28%) having sperm densities of ⩽10 × 10^6^ ml^−1^. There was no statistically significant change at 12 months, with the median now 20 (IQ range 10–39), and 12 patients (31%) having sperm densities of ⩽10 (× 10^6^ ml^−1^). LH, FSH and testosterone were measured before chemotherapy and at 12 months in 34 patients. There were slight rises in both testosterone and gonadotrophin levels at 12 months, the increase being most notable for FSH (median 7.8–9.0 IU l^−1^, *P*<0.001).

### Quality of life

Of 115 eligible patients registered, 102 returned pretreatment questionnaires, 90 of which were within the acceptable window. Compliance with subsequent QoL forms is summarised in Table 3.

### Core questionnaire (EORTC QLQ-C30)

**Figure 1:**
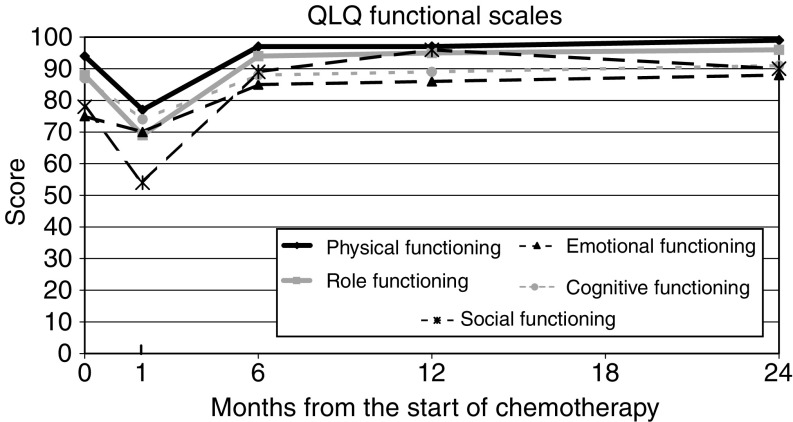
QLQ-C30 functional scales.

**Figure 2:**
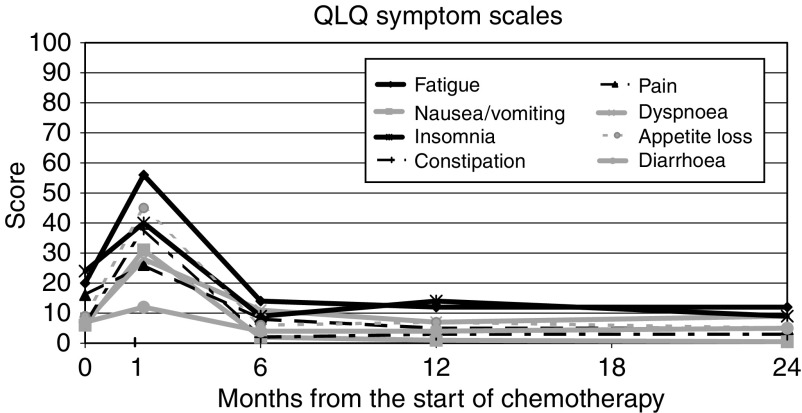
QLQ-C30 symptom scales.

**Figure 3:**
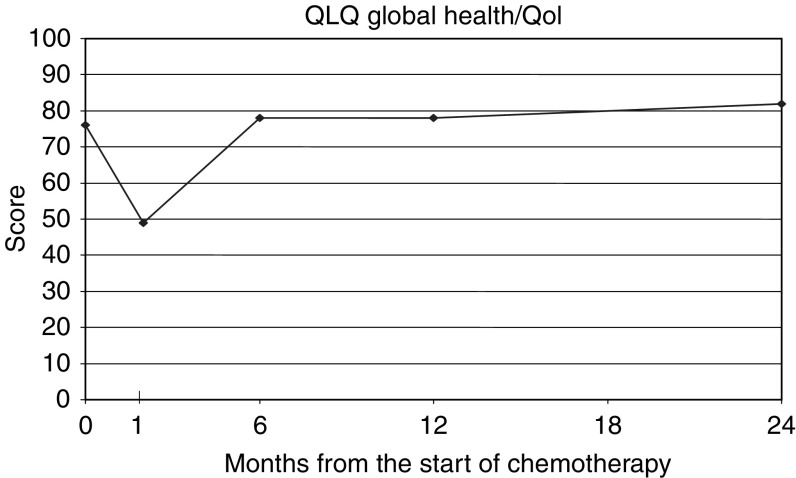
QLQ-C30 – global health scores.

Patterns of change are shown in Table 4 and Figures 1, 2 and 3, which show the mean score (translated to a 0–100 point scale) over all patients for the functional scales (high score=good result), symptom scales (low score=good result), and global health scales (high score=good result) respectively. The figures are based only on patients completing the full set of pre- and post-treatment questionnaires (*n*=41). This represents approximately 46% of the patients with baseline scores, and in these circumstances, analysis of ‘100% compliant’ patients may not be representative of the group as a whole. However, the distribution of scores at specific time points in this group of patients was compared with, and found similar to, the distribution among all patients completing that particular questionnaire.

All the subscales show the same trend, namely a significant decline, relative to baseline, at the end of treatment, followed by recovery, on average to baseline levels or better.

### Testicular tumour questionnaire

In all, 85 patients completed the baseline questionnaire; 41 of these patients also completed each of the subsequent forms up to and including 24 months. Analysis has again focused on these ‘100% compliant’ patients. Table 5 shows the percentage of patients reporting ‘quite a bit’ or ‘very much’ to each of the questions on the testis module at each of the time points. Comparisons of the full range of scores at each time point relative to baseline have been made using the Wilcoxon test for matched data.

Relative to baseline, all individual symptom items produced worse scores at the end of treatment assessment, and those significant at the adjusted significance level of <0.001 being hair loss, pain, numbness or tingling in hands/feet and ringing in the ears. Pain/numbness persisted at the same level of significance at 6 months post-treatment, reducing with time but remaining at the conventional significance level of 5% at 24 months post-treatment. Of the 41 patients completing the full set of questionnaires, five of the 41 (12%) reported ‘quite a bit’ or ‘very much’ pain/numbness at 24 months and the percentage was similar if all 72 patients completing this item of the 24 month questionnaire are considered, irrespective of previous questionnaire compliance (8/72, 11%).

## DISCUSSION

Adjuvant chemotherapy has become a standard of care for men with high-risk stage I testicular teratoma in the UK using two courses of BEP (RCR, 2000). Results of the previous MRC trial TE05 (Cullen *et al*, 1996a) have been updated and with a median follow-up of 7 years show no further recurrences, with one of 103 patients relapsing 6 months after adjuvant chemotherapy. This man had rapidly progressive marker positive liver metastases and died from his disease despite extensive salvage chemotherapy. The 5-year recurrence-free rate in TE05 among those confirmed with germ cell tumours on central pathology review is 99.1% (95% CI 97.3%, 99.9%). Both recurrences in the present trial occurred shortly after adjuvant chemotherapy (3 and 6 months) and both patients received intensive further chemotherapy; one remains alive and disease-free. The 5-year relapse-free rate is 98.3% (95.3, 99.9%). Although these results are barely distinguishable, it is pertinent that the earlier MRC study was in a group of men with a slightly higher expected risk of developing recurrent disease (approximately 50% compared to 35–40% in the current report).

Similar efficacy has been reported for two courses of adjuvant cisplatin-containing chemotherapy by other groups, although the eligibility criteria vary and not all define an agreed ‘high-risk’ group. Pont *et al* (1996) have reported long-term results (median follow-up 79 months) of their study of 29 patients with vascular invasion who received two cycles of adjuvant BEP. One patient relapsed 8 months after treatment and subsequently died from his disease, and another developed a cystic lesion in the retroperitoneal nodes at 27 months; this was resected, found to contain fully differentiated mature teratoma and the patient remains relapse-free with no further treatment. Bohlen *et al* (1999) reported mature results of a study in 58 patients who received two cycles of BEP or PVB (cisplatin, vinblastine, bleomycin). The eligibility criteria for this study defined a group at lower risk (one or more of the risk factors: presence of embryonal carcinoma or vascular invasion, or stage >pT2). With a median follow-up of 93 months, there have been no relapses demonstrating active disease, although one patient again developed a mass in the iliac nodes which contained only teratoma differentiated and was successfully managed with surgery alone. Finally, Abratt *et al* (1994) treated 20 patients ‘at relatively high risk’ with two courses of adjuvant BEP. With a median follow-up of 31 months, no relapses have been reported.

An early fear with the adjuvant approach was that of acquired chemoresistance, leading to poor outcome for those who do relapse. In the literature summary above, six relapses have been reported in 335 patients, and three of the relapsed patients died from their germ cell tumour. These are limited data on which to draw conclusions about acquired chemoresistance, but it would certainly be difficult to improve on the crude cancer-specific survival rate of >99%. It is also noteworthy that all three recurrences in the MRC studies occurred within 6 months of chemotherapy; this is an aggressive pattern of early relapse and would be compatible with an hypothesis of primary rather than acquired chemoresistance.

Since the present study was completed, results of a large randomised MRC/EORTC trial in metastatic germ cell tumours have been published (de Wit *et al*, 2001), establishing three courses of BEP as standard management in good prognosis disease. This is pertinent, as virtually all patients relapsing after postorchidectomy surveillance will do so with good prognosis disease according to the IGCCCG classification (IGCCCG, 1997). The total amount of chemotherapy administered with a policy of adjuvant chemotherapy (200 cycles per 100 high-risk patients) is now considerably higher than a policy of surveillance, with three courses of chemotherapy for the 40% expected to relapse (∼120 cycles per 100 high-risk patients). This is clearly of some economic relevance, but may be of less immediate relevance to the patient, given evidence to suggest that the individual patient's personality and circumstances are the major factors determining whether or not immediate prophylactic treatment is their preference (Cullen *et al*, 1996b). In addition, total amount of chemotherapy is not the only consideration; a significant minority of patients relapsing on surveillance will require retroperitoneal lymph node dissection after chemotherapy. Even so, the trend towards less treatment for metastatic disease gives further motivation to trials such as TE17, aiming to maintain the efficacy of the adjuvant approach, while reducing toxicity.

Although the dose intensity of cisplatin in the BOP schedule is more than with BEP chemotherapy, the bleomycin dose is reduced (30 000 IU × 4 *vs* × 6) and vinca alkaloids have lower efficacy than etoposide (Williams *et al*, 1987). Nevertheless, the current study has an estimated relapse rate at 5 years of 1.7%, and can exclude a relapse rate of more than 5% with probability greater than 95%.

Toxicity of the BOP schedule is different from BEP chemotherapy. Myelosupression is reduced, as judged by day 14 blood counts. However, previous studies of adjuvant treatment have not systematically attempted to record other short- and longer-term toxicities of treatment and make direct comparison of the BOP and BEP schedules difficult. The BOP schedule reduced the near 100% alopecia rate seen with BEP; nevertheless, 59% of patients still reported ‘quite a bit’ (46%) or ‘very much’ (13%) hair loss at the end of treatment. However, only 17% of men with alopecia at the end of treatment reported ‘quite a bit’ or ‘very much’ upset from hair loss. Overall, these results suggest that alopecia is a fairly minor consideration for men currently undergoing adjuvant chemotherapy. This finding may in part depend on the hair fashions current at the time of the study.

Perhaps of more concern is the persistent neuropathy noted as ‘pain, numbness and tingling in hands or feet’ which was recorded as ‘quite a bit’ or ‘very much’ by 37% of men at the end of treatment and 12% of men 2 years later. QoL data has not been collected in any previous trials of adjuvant BEP.^3,13−15^ We therefore chose to compare the BOP data with those of patients receiving three cycles of BEP within the MRC TE20/EORTC 30941 trial of chemotherapy for good prognosis metastatic disease (Fossa *et al*, 2003). For direct comparison, these questionnaire items must be transformed to a 0–100 scale, as for the core questionnaire. In the present study, the mean scores are 41 at the end of treatment and 16 after 2 years of treatment. These are slightly higher than the corresponding figures after three courses of BEP, which were 27 at the end of treatment and 12 after 2 years (with a similar level of compliance with questionnaires). It would be expected that two rather than three courses of BEP would produce less toxicity. Although a nonrandomised comparison, these data strongly suggest that BOP produces more neuropathy than BEP. Reduction of vincristine dosage would be expected to improve tolerance but a further assessment of treatment efficacy would then be required. If undertaken, we consider that a randomised phase II comparison with BEP would be most appropriate and EMG might usefully complement QL or questionnaire-based assessment of peripheral nerve function.

Some impact on high tone hearing loss was detected clinically, this showed slight improvement during follow-up and was paralleled by the testicular cancer module questionnaire responses on hearing loss and tinnitus, which reduced over time back to baseline levels by 24 months. Other QoL indices remained stable or improved with time. No significant changes in testosterone levels or semen analysis were seen, but a slight rise in gonadotrophin levels and a 9% fall in renal function was observed.

Overall the BOP schedule has been shown to be effective adjuvant chemotherapy; however, there are no clearcut advantages over the standard treatment using BEP schedule, except for patients who wish to maximise the chance of avoiding significant alopecia.

## Figures and Tables

**Table 1:** Clinician-assessed toxicity, maximum WHO grades reported during chemotherapy

	**Grade 0**		**Grade 1**		**Grade 2**		**Grade 3**		**Grade 4**	
Alopecia	19	18%	30	28%	36	34%	21	20%	0	—
Neurotoxicity	59	54%	45	41%	6	5%	0	—	0	—
Renal	103	96%	3	3%	0	—	1	1%	0	—
Mucosal	98	91%	6	6%	3	3%	1	1%	0	—
Pulmonary	96	90%	7	7%	4	4%	0	—	0	—
Nausea/vomiting	43	40%	33	31%	19	18%	12	11%	0	—
WBC^a^	78	69%	28	25%	7	6%	0	—	0	—
Platelets^a^	112	99%	0	—	1	1%	0	—	0	—

WHO=World Health Organization; WBC=White blood cells.

a Based on day 14 counts after each cycle.

**Table 2:** Long-term toxicity

**Test**	***N* a**	**Prechemo**	**1 year (± 3 mths) postchemo**	***P*-value for change^b^**
*Audiometry*				
Median loss at 2 kHz (IQ range)	37	5 dB (0–10)	5 dB (0–10)	0.24
Median loss at 4 kHz (IQ range)	38	10 dB (5–15)	10 dB (8–16)	0.24
Median loss at 8 kHz (IQ range)	37	10 dB (5–23)	15 dB (10–30)	0.008
				
*Pulmonary function tests*				
Mean KCO %predicted (s.d.)	23	102 (13)	97 (17)	0.03
Mean FEV %predicted (s.d.)	28	106 (13)	106 (11)	1.00
Mean FVC %predicted (s.d.)	29	110 (13)	109 (11)	0.43
Mean TLC %predicted (s.d.)	22	105 (13)	104 (14)	0.55
				
*Hormones*				
Median LH (IQ range)	34	4.3 (3.6–6.7)	5.7 (4.2–7.5)	0.023
Median FSH (IQ range)	34	7.8 (5.9–9.1)	9.0 (6.9–11.4)	<0.001
Median testosterone (IQ range)	30	14.3 (12.0–19.6)	16.7 (11.3–20.0)	0.57

KCO=transfer coefficient for carbon monoxide; FEV=forced expiratory volume; FVC=forced vital capacity; TLC=total lung capacity; LH=luteinising hormone; FSH=follicle-stimulating hormone.

IQ range=interquartile range; s.d.=standard deviation.

a Number of patients with both baseline and follow-up values.

b Paired *t*-test for pulmonary function test comparisons, Wilcoxon's test elsewhere.

**Table 3:** Summary of compliance with quality of life questionnaires

	**Baseline**	**End of chemo**	**6 Months postchemo**	**12 Months postchemo**	**24 Months postchemo**
Number of forms completed within window	90	87	83	78	73
Number (%) of patients with baseline and follow-up assessment completed within window	90 (100%)	69 (77%)	68 (76%)	66 (73%)	62 (69%)
Cumulative % of patients with full data up to this time point	90 (100%)	69 (77%)	58 (64%)	48 (53%)	41 (46%)

**Table 4:** Summary of QLQ-C30 subscale scores

	**Time point**				
**QLQ-C30 subscales (mean score)**	**Pretreatment**	**End of chemotherapy**	**6 Months postchemo**	**12 Months postchemo**	**24 Months postchemo**
*Function scales*					
Physical	94	77	97	97	99
Role	88	69	94	95	96
Emotional	75	70	85	86	88
Cognitive	87	74	88	89	91
Social	78	54	89	96	90
					
*Global health/Qol*	76	49	78	78	82
					
*Symptom scales*					
Fatigue	20	56	14	12	12
Nausea/vomiting	6	31	2	1	0.5
Pain	16	26	8	5	5
Dyspnoea	7	28	11	7	9
Insomnia	24	40	9	14	9
Appetite loss	9	45	6	7	5
Constipation	7	38	2	3	3
Diarrhoea	7	12	4	4	5
Financial difficulties	26	28	15	11	11

Qol=quality of life.

**Table 5:** Summary data from the testis tumour module

**Testicular tumour module**	**Pretreatment**	**End of treatment**	**6 Months posttreatment**	**12 Months posttreatment**	**24 Months posttreatment**
Have you:	% of patients reporting ‘quite a bit’ or ‘very much’^a^				
… lost any hair?	3%	59%	10%	0%	7%
If yes, been upset by your hair loss?	0%	17%	33%	0%	14%
… had pain, numbness of tingling in your hands and/or feet? 0	3%	37%	24%	13%	12%
… had pale/cold fingers/toes?	0%	15%	12%	7%	7%
… had ringing in the ears?	3%	37%	10%	7%	2%
… had difficulty hearing?	3%	5%	2%	7%	5%
… felt less masculine as a result of your disease or treatment?	5%	15%	7%	7%	5%
… had dry ejaculation during intercourse?	5%	3%	3%	0%	3%
… been interested in sex?	64%	27%	71%	78%	80%^b^
… had difficulty in getting an erection?	5%	15%	2%	13%	5%
… had difficulty in keeping an erection	8%	15%	5%	10%	8%
… had problems with the intensity of your orgasm?	5%	8%	5%	8%	5%
… been sexually active?	41%	20%	54%	54%	58%
If yes, enjoyed sex?	83%	72%	92%	84%	86%
…been worried abut the possibility of being unable to father a child?	18%	13%	7%^b^	10%	2%
… had a satisfying partner relationship?	89%	81%	95%	91%	89%
… feared recurrence of the disease?	32%	37%	29%	18%	20%
… felt that the right treatment decisions were made ?	97%	95%	100%	92%	98%
… felt satisfied with your clinic visits?	90%	95%	93%	97%	90%
… been limited in your hobbies or other leisure pursuits?	31%	51%	7%	3%^b^	2%^b^

a Cells shaded black indicate that a formal comparison with baseline levels was significantly different at the *P*<0.001 level; cells shaded in grey indicate differences at the level 0.001<*P*<0.01.

b Indicates an improvement relative to baseline.

## Appendix A1

The trial Principal Investigator was Professor David Dearnaley, Institute of Cancer Research and Royal Marsden Hospital, Surrey UK. The trial was initiated by the MRC Testicular Tumour Working Party (now the National Cancer Research Institute Testicular Cancer Clinical Studies Group) and coordinated by the MRC Clinical Trials Unit, Cancer Division (formerly the MRC Cancer Trials Office, Cambridge); the trial statistician was Sally Stenning and the senior clinical trial manager was Pat Cook. The centres and clinicians taking part in the trial were as follows:

Norwegian Radium Hospital, Oslo, Norway (20 patients); SD Fossa, N Aass

Beatson Oncology Centre, Glasgow, UK (14 patients); SB Kaye, C Twelves, H Yosef

Birmingham Oncology Centre, Birmingham, UK (9 patients); MH Cullen

Middlesex Hospital, London, UK (nine patients); SJ Harland

Nottingham City Hospital, Nottingham, UK (9 patients); MPJ Sokal

Northern Centre for Cancer Treatment, Newcastle-upon-Tyne, UK (nine patients); JT Roberts, H Lucraft, D Ritchie

Bristol Oncology Centre, Bristol, UK (eight patients); JD Graham

Royal South Hants Hospital, Southampton, UK (6 patients); GM Mead

Addenbrookes Hospital, Cambridge, UK (six patients); MV Williams

Leicester Royal Infirmary, Leicester, UK (six patients); FJ Madden (retired)

Royal Marsden Hospital, Sutton, UK (four patients); DP Dearnaley, A Horwich

Mount Vernon Hospital, Harrow, UK (four patients); GJS Rustin

Clatterbridge Oncology Centre, Liverpool, UK (three patients); PI Clark

Guy's Hospital, London, UK (three patients); PJ Harper

Western General Hospital, Edinburgh (two patients); GCW Howard

Cookridge Hospital, Leeds, UK (one patient); WG Jones (retired)

St James University Hospital, Leeds, UK (one patient); P Selby

## References

[bib1] Abratt RP, Pontin AR, Barnes RD, Reddi BV (1994) Adjuvant chemotherapy for stage I non-seminomatous testicular cancer. South Afr Med J 84(9): 605–607 7530863

[bib2] Bohlen D, Borner M, Sonntag RW, Fey MF, Studer UE (1999) Long-term results following adjuvant chemotherapy in patients with clinical stage I testicular nonseminomatous malignant germ cell tumours with high risk factors. J Urol 161(4): 1148–1152 10081858

[bib3] Christian JA, Huddart RA, Norman A, Mason M, Fossa S, Aass N, Nicholl EJ, Dearnaley DP, Horwich A (2003) Intensive induction chemotherapy with CBOP/BEP in patients with poor prognosis germ cell tumors. J Clin Oncol 21(5): 871–877 12610187 10.1200/JCO.2003.05.155

[bib4] Cullen MH, Billingham LJ, Cook J, Woodroffe CM (1996b Management preferences in stage I non-seminomatous germ cell tumours of the testis: an investigation among patients, controls and oncologists. Br J Cancer 74: 1487–1491 8912550 10.1038/bjc.1996.570 PMC2074781

[bib5] Cullen MH, Stenning SP, Parkinson MC, Fossa SD, Kaye SB, Horwich A, Harland SJ, Williams MV, Jakes R (1996a) Short course adjuvant chemotherapy in high risk stage I non-seminomatous germ cell tumours of the testis (NSGCTT): an MRC study report. J Clin Oncol 14(4): 1106–1113 8648364 10.1200/JCO.1996.14.4.1106

[bib6] de Wit R, Roberts JT, Wilkinson PM, de Mulder PMH, Mead GM, Fosså SD, Cook PA, de Prijck L, Stenning S, Collette L (2001) Equivalence of 3 BEP *versus* 4 cycles and of the 5 day schedule *versus* 3 days per cycle in good prognosis germ cell cancer, a randomized study of the European Organization for Research and Treatment of Cancer Genitourinary Tract Cancer Cooperative Group and the Medical Research Council. J Clin Oncol 19: 1629–1640 11250991 10.1200/JCO.2001.19.6.1629

[bib7] Dearnaley DP (1991) Intensive induction treatment for poor risk patients. In Testicular Cancer, Investigations and Management Horwich A (ed) pp 233–248. London: Chapman & Hall Medical

[bib8] Fayers P, Aaronson N, Bjordal K, Sullivan M (1995) EORTC QLQ-C30 Scoring manual. Brussels: EORTC Study Group on Quality of Life

[bib9] Fossa SD, de Wit R, Roberts JT, Wilkinson PM, de Mulder PHM, Mead GM, Cook P, de Prijck L, Stenning S, Aaronson NK, Bottomley A, Collette L (2003) Quality of life in good prognosis patients with metastatic germ cell cancer: a prospective study of the EORTC Genitourinary group/MRC Testicular Cancer Study Group. J Clin Oncol 21: 1107–1118 12637478 10.1200/JCO.2003.02.075

[bib10] Fossa SD, Moynihan C, Serbouti S (1996) Patients' and doctors' perception of long-term morbidity in patients with testicular cancer clinical stage I – a descriptive pilot study. Supp Care Cancer 4(2): 118–128 10.1007/BF01845761 8673349

[bib11] Freedman LS, Parkinson MC, Jones WG, Oliver RT, Peckham MJ, Read G, Newlands ES, Williams CJ (1987) Histopathology in the prediction of relapse of patients with stage I testicular teratoma treated by orchidectomy alone. Lancet ii(8554): 294–298 10.1016/s0140-6736(87)90889-0 2886764

[bib12] International Germ Cell Cancer Collaborative Group (1997) The International Germ Cell Consensus Classification – a prognostic factor based staging system for metastatic germ cell tumours. J Clin Oncol 15(2): 594–603 9053482 10.1200/JCO.1997.15.2.594

[bib13] Lewis CR, Fossa SD, Mead G, ten Bokkel Huinink W, Harding MJ, Mill L, Paul J, Jones WG, Rodenburg CJ, Cantwell B, Keizer HJ, van oosterom A, Soukop M, Splinter T, Kaye SB (1991) BOP/VIP – a new platinum intensive chemotherapy regimen for poor prognosis germ cell tumours. Ann Oncol 2: 203–211 1710482 10.1093/oxfordjournals.annonc.a057906

[bib14] Merrin C (1980) Combined chemotherapy and cytoreductive surgery for the treatment of advanced testis tumours: 6 years follow-up in 77 patients. Proc ASCO 21: A400

[bib15] Newlands ES, Begent RHJ, Rustin GJS, Parker D, Bagshawe KD (1983) Further advances in the management of malignant teratomas of the testis and other sites. Lancet I(8331): 948–951 10.1016/s0140-6736(83)92079-2 6188011

[bib16] Pont J, Albrecht W, Postner G, Sellner F, Angel K, Holtl W (1996) Adjuvant chemotherapy for high risk clinical stage I nonseminomatous testicular germ cell cancer: long-term results of a prospective trial. J Clin Oncol 14: 441–448 8636755 10.1200/JCO.1996.14.2.441

[bib17] Read G, Stenning SP, Cullen MH, Parkinson MC, Horwich A, Kaye SB, Cook PA (1992) Medical Research Council prospective study of surveillance for stage I testicular teratoma. J Clin Oncol 10: 1762–1768 1403057 10.1200/JCO.1992.10.11.1762

[bib18] Royal College of Radiologists COIN/SIGN guidelines (2000) Management of Adult Testicular Germ Cell Tumours. London, UK: Royal College of Radiologists

[bib19] Wettlaufer JN, Feiner AS, Robinson WA (1984) Vincristine, cisplatin and bleomycin with surgery in the management of advanced metastatic nonseminomatous testis tumours. Cancer 53: 203–209 6197154 10.1002/1097-0142(19840115)53:2<203::aid-cncr2820530203>3.0.co;2-u

[bib20] Williams SD, Birch R, Einhorn LH, Irwin L, Greco FA, Loehrer PJ (1987) Treatment of disseminated germ cell tumours with cisplatin, bleomycin and either vinblastine or etoposide. N Eng J Med 316: 1435–1440 10.1056/NEJM198706043162302 2437455

